# Self-assembly of c-myc DNA promoted by a single enantiomer ruthenium complex as a potential nuclear targeting gene carrier

**DOI:** 10.1038/srep28582

**Published:** 2016-07-06

**Authors:** Qiong Wu, Wenjie Mei, Kangdi Zheng, Yang Ding

**Affiliations:** 1 School of Pharmacy, Guangdong Pharmaceutical University, Guangzhou 510006, China; 2 Traditional Chinese Medicine College, Guangdong Pharmaceutical University, Guangzhou 510006, China

## Abstract

Gene therapy has long been limited in the clinic, due in part to the lack of safety and efficacy of the gene carrier. Herein, a single enantiomer ruthenium(II) complex, Λ-[Ru(bpy)_2_(*p*-BEPIP)](ClO_4_)_2_ (Λ-RM0627, bpy = 4,4′-bipyridine, *p*-BEPIP = 2-(4-phenylacetylenephenyl)imidazole [4,5*f*][1, 10] phenanthroline), has been synthesized and investigated as a potential gene carrier that targets the nucleus. In this report, it is shown that Λ-RM0627 promotes self-assembly of c-myc DNA to form a nanowire structure. Further studies showed that the nano-assembly of c-myc DNA that induced Λ-RM0627 could be efficiently taken up and enriched in the nuclei of HepG2 cells. After treatment of the nano-assembly of c-myc DNA with Λ-RM0627, over-expression of c-myc in HepG2 cells was observed. In summary, Λ-RM0627 played a key role in the transfer and release of c-myc into cells, which strongly indicates Λ-RM0627 as a potent carrier of c-myc DNA that targets the nucleus of tumor cells.

Gene therapy, which encompasses an extensive variety of treatment types, all of which use genetic material to modify damaged cells in an attempt to effect a cure, has long been investigated and considered a promising approach to treat cancer^1^,^2^,^3^. Significant progress has been made in gene therapy, particularly at the animal level, and considerable effort has been exerted to increase the efficiency and safety of this approach^4^,^5^. It is also worth mentioning that the cell nucleus is the major site of DNA replication and transcription, which is the essential target of the gene carrier to affect the functional expression of genes in the cell^6^. Thus, if one gene carrier can target the transfection of a given gene into the nucleus, this might enhance both the transfection efficiency and the stability of gene expression.

Generally, this technology is designed to kill tumor cells directly through introduction of genes called suicide genes; for example, siRNA, miRNA, and mRNA that are targeted to tumor cells^7^,^8^. Moreover, the inhibition of c-myc oncogenic expression can inhibit the proliferation of tumor cells, which is significant because of the critical role played by *c-myc* in regulating the proliferation, division, and metabolism of cells^9^. More importantly, *c-myc* is one of the most crucial transcription factors that regulates the induction of pluripotent stem cells (iPSCs), in which transient expression of the c-Myc transgene plays an important role in the resultant iPSCs^10^,^11^. However, there remain quite significant limitations in the clinical application of not only common gene delivery methods (e.g., nano-liposomes and electroporation), but also the use of retroviral or lentiviral vector systems, due to the lack of an efficient and safe vector system for gene delivery^12^,^13^,^14^,^15^.

To date, considerable effort has been exerted in the design of an efficient gene delivery system. Moreover, non-viral gene vectors, like polymers, liposomes, peptides, chitosan, and nanoparticles, have been extensively investigated. Recently, inorganic materials, such as ultra-small (~2 nm) gold (Au) nanoparticles, have been used to deliver triplex oligonucleotides into the nuclei of cells^16^. Furthermore, transition metal complexes, particularly ruthenium (II) complexes, have been utilized to condense and deliver DNA into cells because of their strong fluorescence, high DNA binding affinity, and low toxicity in biological environments^17^,^18^,^19^,^20^. For example, Kumbhar *et al*., revealed two water-soluble ruthenium (II) polypyridyl complexes as carriers for DNA delivery^21^. Chao *et al*. also developed a luminescent tetranuclear ruthenium (II) complex that served as a tracking non-viral gene vector^22^,^23^,^24^,^25^.

To the best of our knowledge, ruthenium (II) complexes can bind to double-stranded DNA molecules in the intercalating, groove binding, and electrostatic binding modes. Furthermore, the studies by Ji *et al*. showed that ruthenium (II) complexes can bind and stabilize the G-quadruplex DNA structure^26^,^27^. Furthermore, Qu *et al*. found that a zinc-finger-like a single enantiomer metallo-supramolecular complex of NiP, exhibited a single enantiomer selectivity to cancer cells, owing to the target G-quadruplex DNA of telomeres^28^.

Recently, Tan *et al*. achieved many remarkable results in the field of DNA nano-assembly, which might be applied to potential gene carriers, drug delivery systems, and gene-silencing therapy and cellular imaging^3^,^29^,^30^,^31^. By contrast, evidence has previously showed that the guanine-rich sequence of the *c-myc* promoter forms G-quadruplex structures because of three or four guanine bases that are associated in a cyclic array via Hoogsteen hydrogen bonding arrangements^32^,^33^. Studies in our laboratory showed that ruthenium (II) complexes can also facilitate stability of the *c-myc* G-quadruplex DNA^34^,^35^.

With these findings in mind, we hypothesized that a single enantiomer polypyridine ruthenium (II) complex can bind to *c-myc* G-quadruplex DNA and promote the self-assembly of the *c-myc* G-quadruplex DNA. The self-assembled particles carry the DNA into the nuclei of living cells, following which, the DNA is released and functionally expressed as normal. It is anticipated that this approach will help develop a new potential gene carrier system, which might significantly promote the development of gene therapy.

## Results

### Interaction between ***c-myc*** G-quadruplex DNA with **Λ**-RM0627

**Figure 1:**
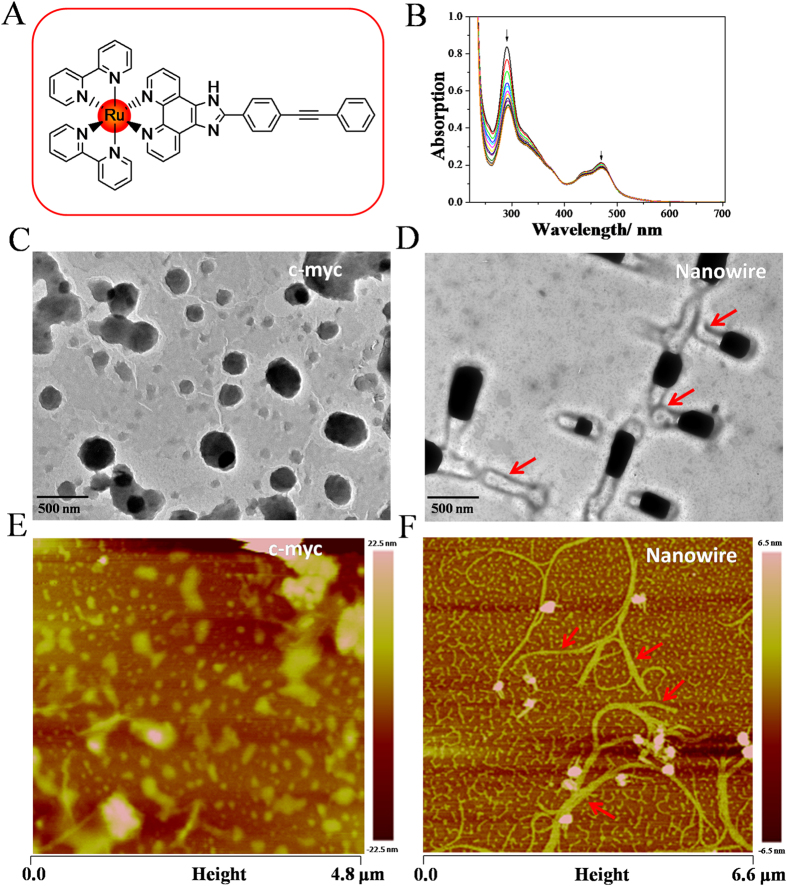
Self-assembly of c-myc G-quadruplex DNA induced by ruthenium complex *Λ*-RM0627. (**A**) Shows the molecular structure of the a single enantiomer ruthenium (II) complex of Λ-RM0627. (**B**) The electronic spectra of Λ*-*RM0627 (20 μM, and 3 mL) were determined following the addition of the *c-myc* G-quadruplex DNA (100 μM) at a rate of 2 μL every 5 minutes. ([*c-myc*] = 0.0667 μM, *n* = 0, 1, 2, 3, … 9, etc.) in Tris-HCl buffer (pH 7.2, containing 100 mM KCl). (**C**) TEM image of free *c-myc* DNA (5 μM) in Tris–HCl KCl solution (pH 7.2) that was air-dried. (**D**) TEM image of *c-myc* DNA (5 μM) self-assembled into nanostructures in the presence of Λ-RM0627 (5 μM) in Tris–HCl KCl buffer (pH 7.2) and air-dried. (**E**) AFM image of *c-myc* DNA (5 μM) in the absence of Λ-RM0627. (**F**) AFM image of the nano-assembly of *c-myc* DNA (5 μM) in the presence of Λ-RM0627.

A single enantiomer ruthenium (II) complex with 2-(4-phenylacetylenephenyl)imidazole[4,5*f*][1,10]phenanthroline (*p-*BEPIP), Λ-[Ru(bpy)_2_(*p-*BEPIP)](ClO_4_)_2_ (Λ-RM0627, bpy = 4,4′-bipyridine) (Fig. 1A), was synthesized in the presence of Pd-Cu catalyst by using microwave-assisted synthesis technology. The binding properties of this complex with *c-myc* G-quadruplex DNA was confirmed by electronic spectra experiments, which is a classic and common method to evaluate the interactions of small molecules with biological macromolecules. According to the electronic spectra experiments, it’s revealed that Λ-RM0627 exhibited strong binding affinity to *c-myc* G4-DNA. This was confirmed by the hypochromism in the electronic absorption of Λ-RM0627 in the presence of *c-myc* G4-DNA. In general, there are characteristic MLCT (metal to ligand charge transfer) absorption in the range of 400–550 nm with a maximum absorption at a wavelength of 470 nm, the IL (i.e., the intraligand charge transfer) absorption in the range of 250–300 nm with a maximum at 290 nm, and A shoulder absorption was observed at a wavelength of 370 nm that could be attributed to the LMCT (i.e., the ligand to metal charge transfer) transition were observed in the electronic spectra of Λ-RM0627 in Tris-HCl buffer (pH 7.2). After G4-DNA was added into the solution, the hypochromism at the MLCT and IL absorption was 14.6 and 38.9%, respectively (Fig. 1B). These data demonstrated that this complexes bind to the *c-myc* G4-DNA with a high affinity^36^.

### DNA self-assembly promoted by **Λ**-RM0627

The self-assembly of the *c-myc* G-quadruplex DNA induced by Λ-RM0627 was confirmed by TEM. We observed that anomalous spherical and compact DNA condensates with diameters of approximately 250 nm were formed by the free*c-myc* G-quadruplex DNA (Fig. 1C). For the *c-myc* G-quadruplex DNA that was incubated with Λ-RM0627, a typical pipeline-like structure with a diameter of approximately 200 nm was observed, indicating that DNA was self-assembled into a nanowire structure in the presence of Λ-RM0627 (Fig. 1D)^36^. Notably, a black area was observed at the terminal and connecting junction of the DNA nanowire^24^.

Atomic force microscopy (AFM) provided additional insights into the self-assembly of the *c-myc* G-quadruplex DNA that was induced by Λ-RM0627. We observed that free*c-myc* DNA formed a number of irregular and spherical DNA particles with sizes of about 50–500 nm and had a height of approximately 22.5 nm (Fig. 1E), which corresponded with the findings obtained by TEM. Thus, we observed a perfect nanowire structure of self-assembled *c-myc* DNA that was promoted by Λ-RM0627. The assembled nanowire had a width of about 25–200 nm and a height of approximately 6.5 nm (Fig. 1F), a typical thickness previously reported for nanowire structures^36^,^37^,^38^,^39^. Moreover, we observed many red points at the terminal and conjunction of the nanowire, which may be the complex Λ-RM0627 confined in the G-quadruplex conformation of DNA^24^.

### Distribution of a single enantiomer ruthenium complex ***Λ***-RM0627

**Figure 2:**
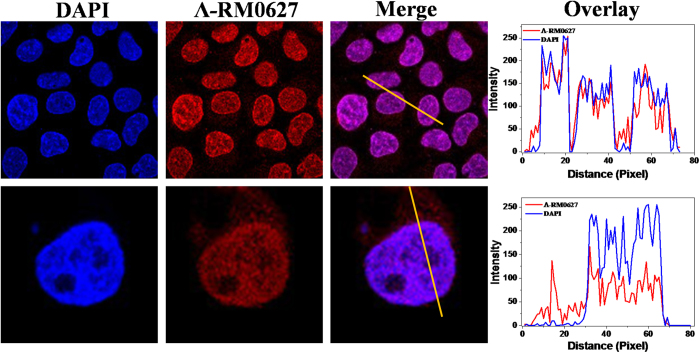
The distribution and localization of ruthenium complex Λ-RM0627. Confocal laser scanning microscopic images of the HepG2 cell nucleus in the presence of Λ-RM0627 (5 μM) treatment for 2 h. The overlay data was analyzed by Image Pro Plus.

This study tested the ability of cellular uptake of a single enantiomer ruthenium complex *Λ*-RM0627 by HepG2 cells. The a single enantiomer ruthenium (II) complex *Λ*-RM0627 (5 μM) was incubated for 2 h, and it was completely absorbed and localized in the nuclei of HepG2 cells (Fig. 2). Interestingly, co-staining with the cyanine dye DAPI, a general nucleic acid stain, shows a clear superposition in localization on nucleus^40^,^41^. In an enlarged image, one can see the emission of *Λ*-RM0627 (red fluorescence) that was merged to blue fluorescence with an overlay rate near 100 percent. These results indicated that complex *Λ*-RM0627 can immediately enter the nucleus.

### Localization of nano-assembly

Given their massive size and low penetrating ability, free DNA cannot enter living cells, which was confirmed in our experiments, wherein the *c-myc* G-quadruplex DNA itself could not be absorbed by HepG2 cells and instead aggregated to the cell surface (Supplementary Fig. 3)^42^. Thus, the FAM (green)-tagged *c-myc* G-quadruplex DNA was used to indicate the location of DNA in cells. After treatment with *Λ*-RM0627, the nanowire self-assembled FAM-*c-myc* G-quadruplex DNA was added to HepG2 cells in fresh DMEM that was supplemented with 10% FBS and incubated with cells for 6 h (Supplementary Fig. 2A). The cellular uptake of DNA was directly observed under confocal laser fluorescence microscopy, and the nuclei of HepG2 cells were stained with 4′,6-diamidino-2-phenylindole (DAPI; blue)^17^,^18^,^22^.

**Figure 3:**
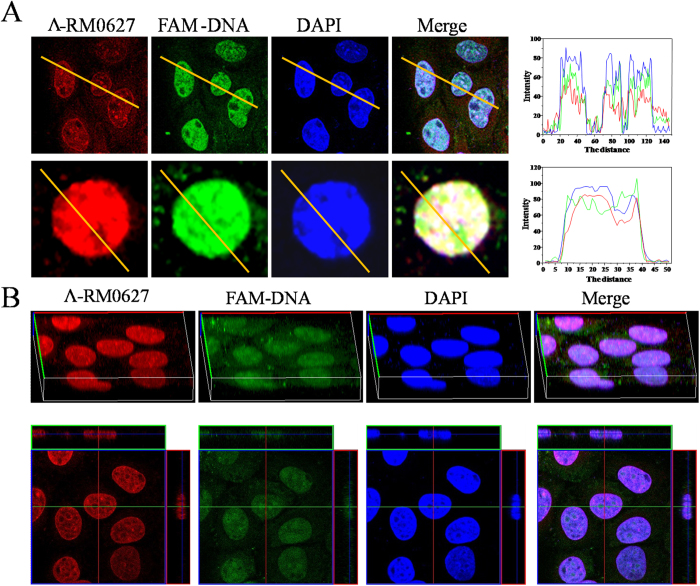
The distribution and localization of the nano-assembly. (**A**) Confocal laser scanning microscopic image of the HepG2 cellular nucleus in the presence of FAM-*c-myc* (5 μM) + *Λ*-RM0627 (5 μM). (**B**) A 3D-tomoscan image of the HepG2 nucleus in the presence of FITC-*c-myc* (5 μM) + *Λ*-RM0627 (5 μM). Red staining shows the a single enantiomer ruthenium (II) complex Λ-RM0627 green staining shows the FAM tagged *c-myc* G-quadruplex DNA and blue staining shows DAPI nuclear counter-staining. The overlay data was analyzed by Image Pro Plus.

We observed that the bright green fluorescence (FAM-*c-myc* G-quadruplex DNA) and red fluorescence (*Λ*-RM0627) were almost at the same site and completely overlaid the blue fluorescence, indicating that the *c-myc* G-quadruplex DNA was successfully absorbed by the cells and localized in the nuclei of the cells (Fig. 3A). In the enlarged image, three-color fluorescence was localized in the same position^43^. Notably, the overlap ratio of DNA, *Λ*-RM0627, and DAPI was approximately 100 percent (Fig. 3B). In addition, in three-dimensional tomoscan imaging, the green fluorescence filled the entire nucleus and was matched to the staining pattern seen for *Λ*-RM0627 and DAPI. This finding indicated that the *Λ*-RM0627 complex could carry FAM-*c-myc* DNA into the nuclei of tumor cells.

### Real-time observation of cellular uptake of nano-assembly

**Figure 4:**
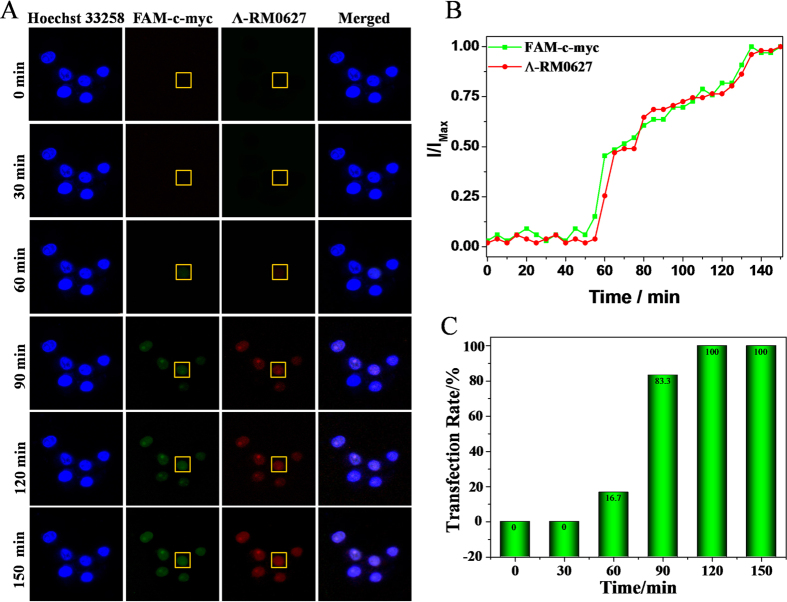
The cellular uptake of the nano-assembly. (**A**) Real-time fluorescence images of HepG2 cells treated with *c-myc* G-quadruplex DNA + Λ-RM0627 (5 μM) for 0, 30, 60, 90, 120, and 150 min. (**B**) Merged position of green and red fluorescence with time. *I*_*Max*_ is the fluorescence seen at 150 min. (**C**)Time-dependent cellular uptake of the *c-myc* G-quadruplex DNA + Λ-RM0627 in HepG2 cells.

To further clarify the possibility of Λ-RM0627 behaving as a potential fluorescence vector to carry specific genes into living cell nuclei, real-time fluorescence observations verified the cellular uptake of the *c-myc* G-quadruplex DNA nanowire that was induced by Λ-RM0627 in living HepG2 cells. The nuclei of HepG2 cells were stained blue by Hoechst 33258 (a typical vital nuclear dye). The DNA particles were then added to the HepG2 cells in DMEM containing 10% FBS. Images were captured every 5 min. After 60 min of incubation, the green fluorescence ascribed to the FAM-*c-myc* G-quadruplex DNA was observed in the nuclei of HepG2 cells. The intensity of green fluorescence and the number of green fluorescently stained cells gradually increased with time (Fig. 4A). Notably, the characterized red fluorescence of Λ-RM0627 was observed at the same site as *c-myc* G-quadruplex DNA in the nuclei.

The time-dependent changes in fluorescence intensity completely corresponded with that of the *c-myc* G-quadruplex DNA during the entire process (Fig. 4B), which indicated that DNA was carried into the nuclei of living cells by Λ-RM0627. After 120 min, the intensity of green fluorescence peaked, and the cellular uptake reached approximately 100 percent (Fig. 4C). These results indicated that the nano-assembled *c-myc* G-quadruplex DNA could transfect cells efficiently as compared with Lipofectamine 2000, which is a classic gene carrier reagent that transfects FAM-*c-myc* G-quadruplex DNA into cells in approximately 6 h and to some extent induces cellular damage (Supplementary Fig. 5)^44^. Moreover, this finding inspired us to develop Λ-RM0627 as an efficient carrier to deliver DNA into the nuclei of living cells for future clinical applications with the aim of accelerating and promoting gene therapy.

### The expression of the nano-assembly

To evaluate the feasibility and effectiveness of this system, the RT-qPCR experiment was completed to define the release and transfection of the *c-myc* G-quadruplex nano-assembly that was induced by Λ-RM0627 in HepG2 cells. Next, data that was obtained by RT-qPCR showed that the ruthenium complex delivered c-myc into living cells and enabled normal expression of the c-myc gene. With increasing concentrations of the nano-assembly, the expression of the c-myc gene was clearly enhanced in HepG2 cells. When the nanoassembly system concentration was 5 μM, the expression of the c-myc gene was increased by approximately 17.5 percent as compared to 10.9 percent of Lipofectamine 2000, in which the results indicated that the nano-assembly of c-myc DNA could not only enter living cells quickly, but could also release and express c-myc normally^45^. These results were agreement with the researches of Chao group, which the ruthenium complex can deliver the DNA into the cells and the gene can express normally in cells^24^.

### The cytotoxicity of the nano-assembly

**Figure 5:**
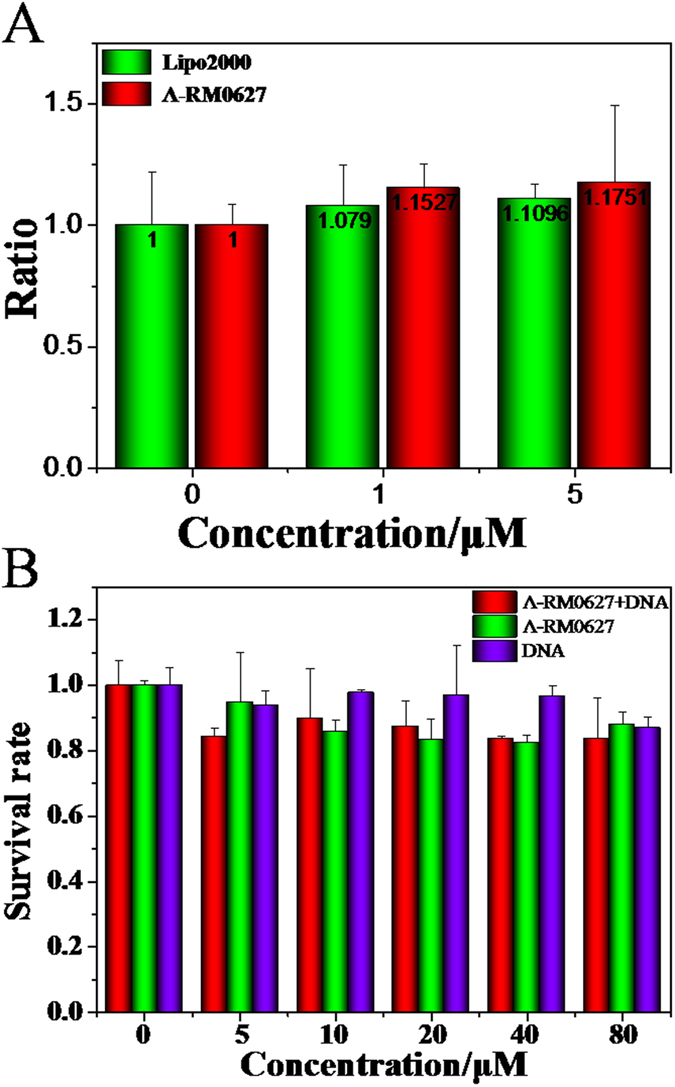
Expression of the c-myc gene and the toxicity of the c-myc nano-assembly induced by Λ-RM0627. (**A**) Regulation of the expression of c-myc DNA delivered byLipofectamine 2000 and ruthenium complex Λ-RM0627 at the RNA level. [DNA/Lipofectamine 2000] = 1 μg/10 μL; [DNA]: [Λ-RM0627] = 1:1 [DNA] = (0, 1 and 5 μM). (**B**) The cytotoxicity of the a single enantiomer ruthenium (II) complex *Λ*-RM0627, free *c-myc DNA* and Λ-RM0627 +*c-myc* DNA is shown. All data were obtained from three independent experiments and were presented as the mean ± SD. P < 0.05 vs untreated control. Error bars represent the mean standard deviation.

The cytotoxicity of Λ-RM0627, free *c-myc* DNA and the nano-assembly were evaluated by MTT with HepG2 cells, respectively. According to the MTT assay, it was found that Λ-RM0627 and free *c-myc* DNA displayed no significant inhibitory effect on growth, in which about 90 percent of cells remained viable (Fig. 5B). Moreover, these results indicated that the nano-assembly exhibited low cytotoxicity against HepG2 cells, in which the cell survival rate still exceeded 80 percent when the concentration reached 80 μM^17^.

## Discussion

In this regard, a novel approach has been constructed to develop a potent gene carrier system to target the nucleus of tumor cells. The current technology has focused on the nanowire structure formed from the self-assembly of c-myc DNA that was promoted by a novel a single enantiomer ruthenium (II) complex, Λ-RM0627. The nanowire of the assembled c-myc G-quadruplex DNA was rapidly absorbed and localized to the nucleus, and consequently, the expression of the c-myc gene was up-regulated. To our knowledge, this study is the first attempt to utilize a single enantiomer ruthenium (II) complexes to carry DNA into the nuclei of living tumor cells. Compared with Lipofectamine 2000 and other investigated gene delivery systems, we were impressed by the rapid and high transfection rate that was displayed by the nano-assembled *c-myc* DNA-Λ-RM0627 system, in addition to its visibility in the nuclei of living cells.

**Figure 6:**
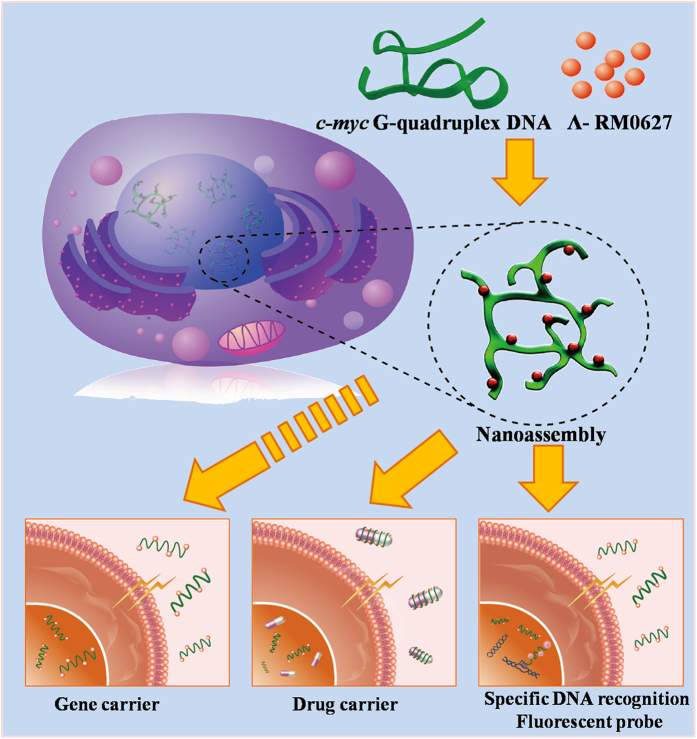
The potential application of the DNA nano-assembly that was promoted by Λ-RM0627. Illustration of the *c-myc* G-quadruplex DNA nano-assembly that was induced by the a single enantiomer ruthenium (II) complex to carry DNA into the nuclei of living cells. Also shown is the potential application of the nano-assembly system as a drug and gene carrier and specific DNA recognizing fluorescent probe.

Given these advantages, the developed DNA carrier may provide an invaluable platform for gene delivery applications^17^, and could catalyze the fundamental development of a single enantiomer ruthenium (II) complexes as carriers in gene delivery and clinical therapy^3^. In summary, we developed a novel method by using the a single enantiomer ruthenium (II) complex to promote the self-assembly of the c-myc G-quadruplex DNA that could carry the gene into the nuclei of viable tumor cells. The proposed method could be easily applied to gene therapeutic applications in the near future (Fig. 6).

## Methods

### Materials

All reagents and solvents were purchased commercially and used without further purification unless specifically noted. Distilled water was used in all experiments. The *c-myc* pu22 DNA (5′-TGGGGAGGGTGGGGAGGGTGGGGAAGG-3′) and FITC labelled *c-myc* pu22 DNA (5′-FAM-TGGGGAGGGTGGGGAGGGTGGGGAAGG-3′) were purchased from Sangon Biotech (Shanghai) Co., Ltd. The G-quadruplex conformation was formed by denaturation at 90 °C for 5 min followed by denaturation at 4 °C for 24 h, as stipulated by previously published methods^43^. All aqueous solutions were prepared in double-distilled water. Fetal bovine serum (FBS), Dulbecco’s Modified Eagle Medium (DMEM), penicillin and streptomycin were purchased from Gibco/Life Technologies (Grand Island, NY). Hoechst 33258 and DAPI were purchased from Beyotime Biotechnology. Lipofectamine 2000 was purchased from Invitrogen.

### Instruments

These complexes were synthesized by using an Anton Paar monowave 300 microwave reactor, which was set at 2450 MHz at an initiating single mode power setting (Biotage). The ^1^H NMR, ^13^C NMR and ^1^H ^1^H COSY spectra were recorded in *d*_6_-DMSO solution on a Bruker DRX 2500 spectrometer, and ESI-MS spectra were obtained in methanol on an Agilent 1100 ESI-MS system that was operated at room temperature. UV-vis absorption spectra were recorded on a Shimadzu UV-2550 spectrophotometer using 1 cm path-length quartz cuvettes (3 mL). Circular dichroism (CD) spectra were measured on a Jasco J-810 spectropolarimeter.Cellular localization and real-time fluorescence imaging experiments were measured using an LSCM510 Meta Duo Scan(Carl Zeiss, Germany). The nanostructures were characterized by transmission electron microscopy (TEM, model TECNAI 10) and atomic force microscopy (AFM) (Bruker, Dimension FastScan^TM^).

### Preparation of ***Λ***-RM0627

*Λ*-RM0627 was synthesized from *Λ*-[Ru(bpy)_2_(*p*-BrPIP)](ClO_4_)_2_ with phenylacetylene by the Sonogashira coupling reaction, which was followed according to previously published literaturebut with some modifications^46^.

### Self-Assembly of c-myc G-quadruplex DNA with **Λ**-RM0627

The mixed solution of DNA (50 μM) and Λ-RM0627 (50 μM) were incubated for three days, following which the mixed solution at a volume of 100 μL was added to a copper wire mesh and naturally volatilized for 2 h. An image of the sample was captured by transmission electron microscopy (TEM) using a TECNAI 10 TEM. The mixed solution at a volume of 10 μL was then removed and added to a mica plate and naturally volatilized for 2 h. Again, an image was captured by atomic force microscopy (AFM) (Bruker, Dimension FastScan^TM^).

### Distribution and location of the nano-assembly of c-myc G-quadruplex DNA with **Λ**-RM0627

HepG2 cells were cultured in DMEM culture medium that was supplemented with 10% fetal bovine serum (FBS) at 37 °C and 5% CO_2_. After being digested by trypsin-EDTA solution, the cells were counted and divided into two parts. Each part (5 × 10^4^ cells) were seeded onto cover slips (18-mm diameter) and allowed to adhere for 12 h before changing the culture medium to DMEM with the nano-assembly of the *c-myc* G-quadruplex DNA (5 μM) with *Λ*-RM0627 (5 μM). The cells were incubated with the complex for 6 h at 37 °C under 5% CO_2_ followed by carefully washing the cells with PBS buffer, then fixed by 4% paraformaldehyde and stained with DAPI. There are no obvious increase membrane permeabilisation under the combination of Λ-RM0627 and laser light in the current laser intensity was observed. The pictures were captured by confocal laser microscopy (Zeiss, LSM 510).

### Cellular uptake of the c-myc G-quadruplex DNA nano-assembly with ***Λ***-RM0627 observed in real-time

The HepG2 cells (at a density of 5 × 10^4^ cells) were seeded onto cover slips (18-mm diameter) and allowed to adhere for 12 h. Hoechst 33258 (10 nM) was then added to the living cells and incubated for 30 min to stain the cell nucleus. Next, the culture medium was changed to DMEM solution including the *c-myc* G-quadruplex DNA (5 μM) nano-assembly with *Λ*-RM0627 (5 μM). Images were then captured by confocal laser microscopy about every 5 min for 150 min.

### RT-qPCR analysis

The nanowire system (50 μM) of *Λ*-RM0627 and *c-myc* (sterile KCl Tris-HCl buffer)pre-incubation for 3 days were diluted in DMEM with 10% FBS to 5 μM. HepG2 cells were incubated with the nanowire system (5 μM) at 37 °C for 24 h. The primers were purchased from BGI, Trizol was purchased from Invitrogen and quantitative PCR using SYBR green system was purchased from Takara. Total RNA was isolated using Trizol according to the recommendation of the manufacturer. Up to 2 μg of the total RNA from each sample was reverse transcribed using oligo (dT) primers at 37 °C for 90 min^47^. The relative mRNA levels were evaluated by quantitative PCR using a SYBR green PCR kit. The signals were normalized to β-actin as an internal control. The quantity of*c-myc*in each BC, relative to the average expression in 40 NATs, was calculated using the following equation: *RQ* = 2^−ΔΔ*CT*^, where ΔΔ*C*_*T*_ = (*C*_*T c-myc*_ − C_*T* β-actin_) _S_ − (*C*_*T c-myc*_ − C_*T* β-actin_) Mean_C_. The primer sequences are listed in the Supporting Information.

## Additional Information

**How to cite this article**: Wu, Q. *et al*. Self-assembly of c-myc DNA promoted by a single enantiomer ruthenium complex as a potential nuclear targeting gene carrier. *Sci. Rep.* **6**, 28582; doi: 10.1038/srep28582 (2016).

## Supplementary Material

Supplementary Information
