# Supplementary material for: Self-assembly of c-myc DNA promoted by a single enantiomer ruthenium complex as a potential nuclear targeting gene carrier
Source: Sci Rep. 2016 Jul 6;6:28582. doi: 10.1038/srep28582 (PMC4933878; doi:10.1038/srep28582)
Supplement: Supplementary Information [file srep28582-s1.doc]

**Self-assembly of c-myc DNA promoted by a single enantiomer ruthenium complex as a potential nuclear targeting gene carrier**

Qiong Wu^1^, Wenjie Mei^1^, Kangdi Zheng^2^ & Yang Ding^1^

^1^ School of Pharmacy, Guangdong Pharmaceutical University, Guangzhou 510006, China. ^2^Traditional Chinese Medicine College, Guangdong Pharmaceutical University, Guangzhou 510006, China. Correspondence and requests for materials should be addressed to W.-J.M. (email: wenjiemei@126.com)

CONTENTS

[**1. EXPERIMENTAL SECTION** 2](#__RefHeading___Toc433201615)

[1.1 Material and Methods 2](#__RefHeading___Toc433201616)

[1.2 Instrument 2](#__RefHeading___Toc433201617)

[1.3 Synthesis of *Λ*-[Ru(bpy)_2_(p-PBE)](ClO_4_)_2_ 3](#__RefHeading___Toc433201618)

[1.4 The characterization of *Λ*-RM0627 4](#__RefHeading___Toc433201622)

[1.5 The cellular localization of *Λ-*RM0627 5](#__RefHeading___Toc433201623)

[1.6 The cellular uptake of *c-myc* DNA 5](#__RefHeading___Toc433201624)

[1.7 The transfection of *c-myc* DNA by Lipo 2000 5](#__RefHeading___Toc433201625)

[1.8 The TEM figures of self-assembling *c-myc* DNA by *Λ*-RM0627 6](#__RefHeading___Toc433201626)

[1.9 The AFM figures of self-assembling *c-myc* DNA by *Λ*-RM0627 6](#__RefHeading___Toc433201627)

[1.10 The cell survival rate of self-assembling *c-myc* DNA 6](#__RefHeading___Toc433201628)

[1.11 RT-qPCR analysis 7](#__RefHeading___Toc433201629)

[**2. RSULTS** 8](#__RefHeading___Toc433201630)

[**3. REFERENCES** 13](#__RefHeading___Toc433201631)

# 1. EXPERIMENTAL SECTION

## 1.1 Material and Methods

All reagents and solvents were purchased commercially and used without further purification unless specially noted. Distilled water was used in all experiments. The *c-myc* G-quadruplex DNA (5′-TGGGGAGGGTGGGGAGGGTGGGGAAGG-3′) and

FITC labelled *c-myc* pu22 DNA (5′-FITC-TGGGGAGGGTGGGGAGGGTGGGGAAGG-3′) were purchased from Sangon Biotech (Shanghai) Co., Ltd. The c-myc DNA was resolve in Tris-HCl KCl buffer solution for 100 μM and the G-quadruplex conformation was formed by denaturation at 90 °C for 5 min followed by renaturation at 4 °C for 24 h, then we pre-incubation of the complex and the DNA in the Tris-HCl KCl buffer solution (germ-free) 3 days and add into DMEM medium with 10% FBS, which the Tris-HCl buffer is consisting of Tris (10 mM) and KCl (100 mM), and the pH value was adjusted to 7.4 by HCl (0.1 mol), as stipulated by previously published methodswere purchased from Sangon Biotech (Shanghai) Co., Ltd. The G-quadruplex conformation was formed by denaturation at 90 °C for 5 min followed by denaturation at 4 °C for 24 h, as stipulated by methods in other studies^1^. All aqueous solutions were prepared with double-distilled water. Fetal bovine serum (FBS), Dulbecco’s Modified Eagle Medium (DMEM), and penicillin/streptomycin were purchased from Gibco by Life Technologies (Grand Island, NY). Hoechst 33258 and DAPI were purchased from Beyotime Biotechnology. Lipo2000 was purchased from Invitrogen.

## 1.2 Instrument

These complexes were synthesized by using Anton Paar monowave 300 microwave reactor (an Initiator single mode microwave cavity at 2450 MHz (Biotage)). The ^1^H NMR, ^13^C NMR and ^1^H ^1^H COSY spectra were recorded in *d*_6_-DMSO solution on a Bruker DRX 2500 spectrometer, and ESI-MS spectra were obtained in [methanol](http://dict.youdao.com/search?q=methanol&keyfrom=e2ctranslation) on Agilent 1100 ESI-MS system operating at room temperature. UV-vis absorption spectra were recorded on a Shimadzu UV-2550 spectrophotometer using 1 cm path length quartz cuvettes(3 mL). Circular dichroism (CD) spectra were measured on a Jasco J-810 spectropolarimeter. Cellular localization and real-time fluorescence images experiments were measured by LSCM510 Meta Duo Scan (Carl Zeiss, Germany). The nanostructure were characterized by transmission electron microscopy (TEM) (TECNAI 10) and atomic force microscopy (AFM) (Bruker, Dimension FastScan^TM^)

## 1.3 Synthesis of *Λ*-[Ru(bpy)_2_(p-PBE)](ClO_4_)_2_

**Figure S1** Micorwave-assisted synthesis route of chiral ruthenium(II) complex ***Λ*-RM0627**.

### 1.3.1 Synthesis of 2-(4-bromophenyl)imidazo[4,5*f*][1,10]phenanthroline (*p*-BrPIP)

2-(4-bromophenyl)imidazo[4,5*f*][1,10] phenanthroline (*p*-BrPIP) was prepared by a similar method as the literatures with some modifications. In general Phenanthroline-5,6-dione (347 mg, 1.6 mmol),  4-Bromobenzaldehyde (294 mg, 1.6 mmol) and ammonium acetate (2.53 g) was dissolved in 20 mL acetic acid and the mixture was irradiated under microwave at 110 ^o^C for 30min. Then 20 mL of water was added and the pH value was adjusted to 7.0 by ammonia water at room temperature. A large number of yellow precipitate were obtained after filtration, and then, it was dried under vacuum. The products were purified silica gel chromatography using ethanol as eluent to give the title compound, yield of 93.2%.

### 1.3.2 Synthesis of *Λ*-[Ru(bpy)_2_(*p*-BrPIP)](ClO_4_)_2_ (*Λ*-1)

*Λ*-1 was synthesized following the literature but with some modifications^2^. A mixture of Λ-[Ru(bpy)_2_(py)_2_][o,o’-dibenzoyl-D-tartrate]∙12 H_2_O (520 mg, 0.4 mmol), p-BrPIP (225 mg, 0.6 mmol) and ethylene glycol (54 mL) was refluxed for 8 h under argon. The cooled reaction mixture was diluted with water. Saturated aqueous ammonium sodium perchlorate solution was added under vigorous stirring, and filtered. The dark red solid was collected and washed with small amounts of water and diethyl ether, then dried under vacuum, and purified by Al_2_O_3_ column chromatography on alumina with acetonitrile/toluene (2 :1 v/v) as eluent. The solvent was removed under reduced pressure and red microcrystals were obtained; yield: 73.0 %. ESI-MS (in CH_3_CN, m/z): 888.6 ([M-ClO_4_]^+^), 789.1 ([M-2ClO_4_-H]^+^), 395.3 ([M-2ClO_4_]^2+^).

### 1.3.3 Synthesis of *Λ*-[Ru(bpy)_2_(p-PBE)](ClO_4_)_2_ (*Λ*-RM0627)

*Λ*-RM0627 was synthesized following the literatures but with some modifications^2^. In general, Λ-1 (130 mg, 0.125 mmol) and phenylacetylene (0.09 mL, 0.625 mmol) was dissolved in dry CH_3_CN (15.0 mL), Pd(PPh_3_)_2_Cl_2_ (3.5 mg, 0.005 mmol), CuI (2 mg, 0.010 mmol) and dry Et_3_N (0.02 mL) were then added under N_2_ atmosphere. The reaction mixture was irradiated by microwaves for 30 min at 140 °C. After filtration and evaporation of the solvent, the residue was purified by flash Al_2_O_3_ column chromatography by using CH_3_CN as elute, yields, 48.2%.

## 1.4 The characterization of *Λ*-RM0627

The structure of ***Λ*-RM0627** were characterizated by ESI-MS spectra, ^1^H NMR spectra, ^1^H ^1^H COSY spectra, ^13^C NMR spectra, and CD spectra. ESI-MS (in CH_3_CN, m/z): 809.3 ([M-2ClO_4_-H]^+^), 405.3 ([M-2ClO_4_]^2+^). UV-vis [λ(nm), ε (M^-1^cm^-1^) (in 5% DMSO/H_2_O]: 469.5 (19800), 290.5 (77200), 264 (35700). CD[λ_max_ (nm), in 5% DMSO/H_2_O]: +298. ^1^H NMR (500 MHz, d^6^-DMSO, ppm) δ 9.07 (d, J = 8.1 Hz, 2H) , 8.90 (d, J = 8.2 Hz, 2H) , 8.86 (d, J = 8.2 Hz, 2H) , 8.41 (d, J = 8.3 Hz, 2H), 8.25 (m, 2H) , 8.12 (t, J = 7.4 Hz, 2H), 7.98 (d, J = 4.4 Hz, 2H), 7.88 (t, 6H), 7.78 (d, J = 7.9 Hz, 2H), 7.6-7.53 (dd, 6H), 7.47 (dd, J = 4.8, 1.7 Hz, 2H), 7.37 (t, J = 6.5 Hz, 2H). ^13^C NMR (126 MHz, d^6^-DMSO, ppm) δ 157.20 (s), 151.79 (s), 144.74 (s), 138.21 (s), 132.28 (s), 131.84 (s), 130.60 (s), 129.30 (s), 128.26 (s), 127.01 (s), 126.07 (s), 124.87 (s), 122.77 (s), 89.32 (s).

The further studies of the fluorescence intensity of *Λ*-RM0627 with little changes when the addition of same volume water gradually in the solution, which indicated that the changes of *Λ*-RM0627 with FITC-*c-myc* ascribed to the interaction of two molecules and the energy transfer from *Λ*-RM0627 to FITC-*c-myc* (Figure S2A). This conclusion was further confirmed by the fluorescence intensity of FITC-*c-myc* increasing step by step when the addition of same volume FITC-*c-myc* gradually in the water (Figure S2B)

**Self-Assembly of c-myc G-quadruplex DNA with *Λ*-RM0627.**

The mixed solution of DNA (50 μM) and *Λ-*RM0627 (50 μM) were incubated for three days, following which the mixed solution at a volume of 100 μL was added to  a copper wire mesh and naturally volatilized for 2 h. An image of the sample was captured by transmission electron microscopy (TEM) using a TECNAI 10 TEM. The mixed solution at a volume of 10 μL was then removed and added to a mica plate and naturally volatilized for 2 h. Again, an image was captured by atomic force microscopy (AFM) (Bruker, Dimension FastScan^TM^).

## 1.5 The cellular localization of *Λ-*RM0627

The HepG2 cells were grown in DMEM medium supplemented with 10% Fetal Bovine Serum (FBS) at 37℃ and 5% CO_2_. After being digested by Trypsine EDTA solution, the cells were counted and divided into two parts^3,4^. Each part (2×10^6^ cells) were seeded in 75 cm^2^ culture flasks and allowed to adhere for 12 h before changing the culture medium to DMEM solution with ruthenium(II) complex ***Λ-*RM0627** (5 μM), respectively. The cells were incubated with the complex for 2h at 37℃ under 5% CO_2_ followed by carefully washing cells with PBS solution and then stained with DAPI. The pictures wrer captured by confocal laser microscopy (Zeiss, LSM 510)..

## 1.6 The cellular uptake of *c-myc* DNA

The HepG2 cells were grown in DMEM medium supplemented with 10% Fetal Bovine Serum (FBS) at 37℃ and 5% CO_2_. Each part (2×10^6^ cells) were seeded in 75 cm^2^ culture flasks and allowed to adhere for 12 h before changing the culture medium to DMEM solution with FITC-DNA (c-myc pu22) (5 μM). The cells were incubated with FITC-DNA for 6 h at 37℃ under 5% CO_2_. The pictures wrer captured by fluoresence microscopy (EVOS, FLoid™ Cell Imaging Station).

## 1.7 Distribution and location of the nano-assembly of c-myc G-quadruplex DNA with Λ-RM0627.

The HepG2 cells were cultured in DMEM culture medium that was supplemented with 10% fetal bovine serum (FBS) at 37℃ and 5% CO_2_. After being digested by trypsin-EDTA solution, the cells were counted and divided into two parts. Each part (5×10^4^ cells) were seeded onto cover slips (18-mm diameter) and allowed to adhere for 12 h before changing the culture medium to DMEM with the nano-assembly of the *c-myc* G-quadruplex DNA (5 μM) with *Λ*-RM0627 (5 μM). The cells were incubated with the complex for 6 h at 37 ℃ under 5% CO_2_ followed by carefully washing the cells with PBS buffer and then stained with DAPI. The pictures were captured by confocal laser microscopy (Zeiss, LSM 510).

## 1.8 Cellular uptake of the c-myc G-quadruplex DNA nano-assembly with Λ-RM0627 observed in real-time.

The HepG2 cells (at a density of 5×10^4^ cells) were seeded onto cover slips (18-mm diameter) and allowed to adhere for 12 h. Hoechst 33258 (10 nM) was then added to the living cells and incubated for 30 min to stain the cell nucleus. Next, the culture medium was changed to DMEM solution including the *c-myc* G-quadruplex DNA (5 μM) nano-assembly with *Λ*-RM0627 (5 μM). Images were then captured by confocal laser microscopy about every 5 min for 150 min.

## 1.9 RT-qPCR analysis

The nanowire system (50 μM) of *Λ*-RM0627 and *c-myc* (sterile KCl Tris-HCl buffer) pre-incubation for 3 days were diluted in DMEM with 10% FBS to 5 μM. HepG2 cells were incubated with the nanowire system (5 μM) at 37 ^o^C for 24 h. The primers were purchased from BGI, Trizol was purchased from Invitrogen and quantitative PCR using SYBR green system was purchased from Takara. Total RNA was isolated using Trizol according to the recommendation of the manufacturer. Up to 2 μg of the total RNA from each sample was reverse transcribed using oligo (dT) primers at 37 °C for 90 min ^43^. The relative mRNA levels were evaluated by quantitative PCR using a SYBR green PCR kit. The signals were normalized to β-actin as an internal control. The quantity of *c-myc* in each BC, relative to the average expression in 40 NATs, was calculated using the following equation: *RQ*= 2^−ΔΔ^*^CT^*, where ΔΔ*C_T_* = (*C_T_* *_c-myc_* – C*_T_* _β-actin_) _S_ − (*C_T_* *_c-myc_* – C*_T_* _β-actin_) Mean_C_. The primer sequences are listed in the Supporting Information.

c-myc Forward, 5’-AAG CCA CAG CAT ACA TCC- 3’

c-myc Reverse, 5’-CAA GAC TCA GCC AAG GTT- 3’

β-actin Forward, 5’- TGC GTG ACA TTA AGG AGA A- 3’

β-actin Reverse, 5’- AAG GAA GGC TGG AAG AGT- 3’

## 1.10 The transfection of *c-myc* DNA by Lipo 2000

The HepG2 cells were grown in DMEM medium supplemented with 10% Fetal Bovine Serum (FBS) at 37℃ and 5% CO_2_. Each part (2×10^6^ cells) were seeded in 75 cm^2^ culture flasks and allowed to adhere for 12 h before changing the culture medium to DMEM solution with FITC-DNA (c-myc pu22) and Lipo 2000. The cells were incubated with FITC-DNA for 6 h at 37℃ under 5% CO_2_. The pictures were captured by fluoresence microscopy (EVOS, FLoid™ Cell Imaging Station).

## 1.11 The TEM figures of self-assembling *c-myc* DNA by *Λ*-RM0627

The nanowire system (50 μM) of *Λ*-RM0627 and *c-myc* (sterile KCl Tris-HCl buffer) pre-incubation for 3 days. Then remove the mixed solution 100 μL to  copper wire mesh and natural volatilize 2 h. The sample was captured picture by transmission electron microscopy^5,6^ (TEM) (TECNAI 10).

## 1.12 The AFM figures of self-assembling *c-myc* DNA by *Λ*-RM0627

The nanowire system (50 μM) of *Λ*-RM0627 and *c-myc* (sterile KCl Tris-HCl buffer) pre-incubation for 3 days. Then remove the mixed solution 10 μL to mica plate and natural volatilize 2 h. The sample was captured picture by atomic force microscopy^7,8^ (AFM) (Bruker, Dimension FastScan^TM^).

## 1.13 The cell survival rate of self-assembling *c-myc* DNA

The cytotoxicity of the chiral ruthenium(II) complex *Λ*-RM0627, c-myc pu22 DNA and *Λ*-RM0627 +c-myc pu22 DNA toward human liver cancer HepG2 cells, has been studied by the MTT. All the cells growing in log phase were seeded into 96-well cell culture plate at 1×10^4^/well and allowed to adhere for 24 h. After serial dilutions of the ruthenium(II) complexes had been added, the cells were incubated for an additional 24 h at 37℃ under 5% CO_2_. On completion of the incubation, MTT solution [MTT working solution, 5 mg/mL phosphate-buffered saline (PBS), 20 μl per well was added, and the cells were incubated for 4 h. Then, the medium was aspirated and replaced with 150μl of DMSO per well to dissolve the formazan^9^. The optical density of each well was measured using a microplate reader at a wavelength of 490 nm. The half-maximal inhibitory concentration (IC_50_) was determined by a plot of the viability versus the dose used to treat the cells. All data were from at least three independent experiments and are expressed as the mean ± the standard deviation.The following formula was used tocalculate the viability of cell growth:

Viabilitye(%) = (mean of Absorbance value of treatment group / mean Absorbance

value of contro)﹒100

# 2. RSULTS

A


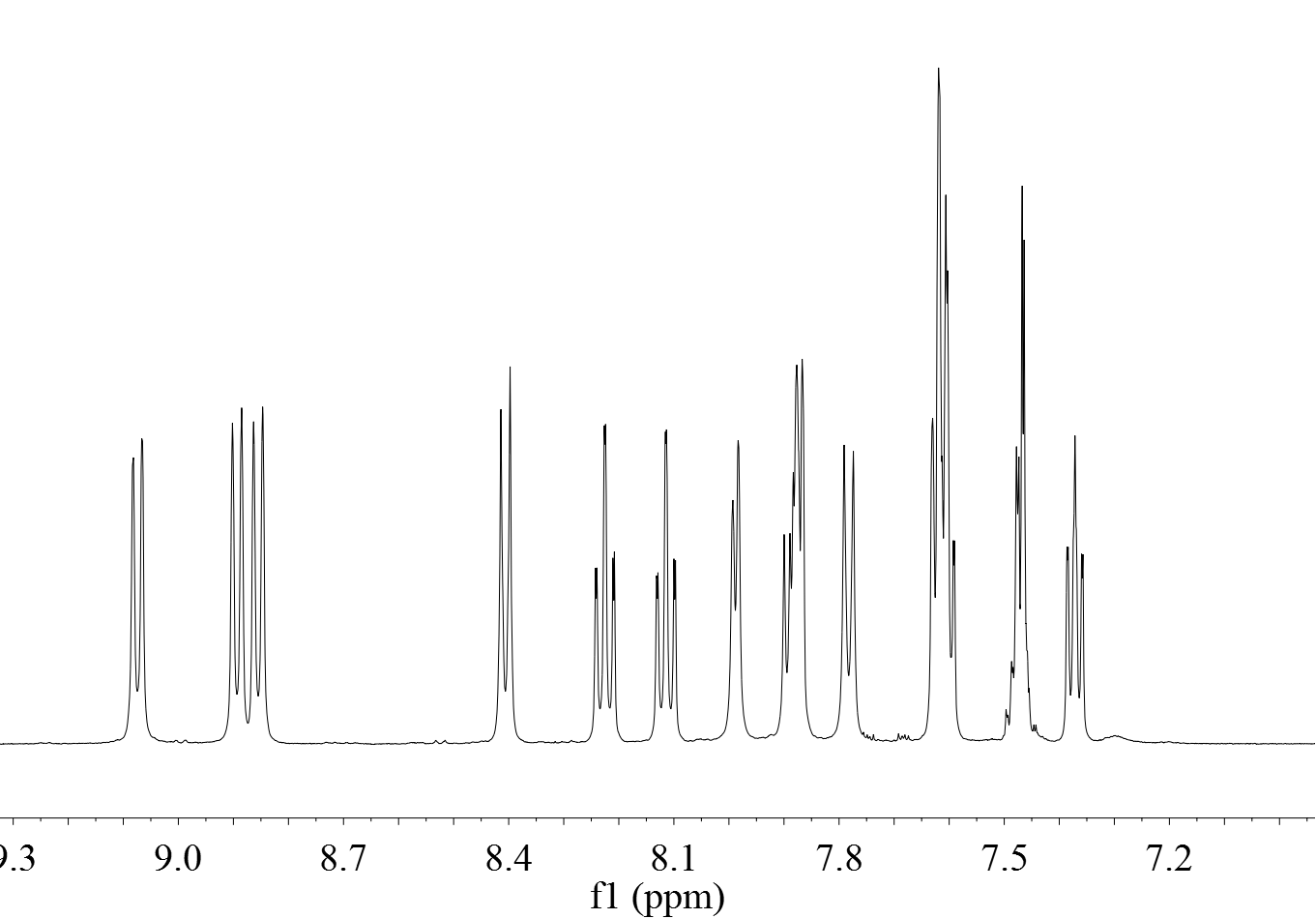


B


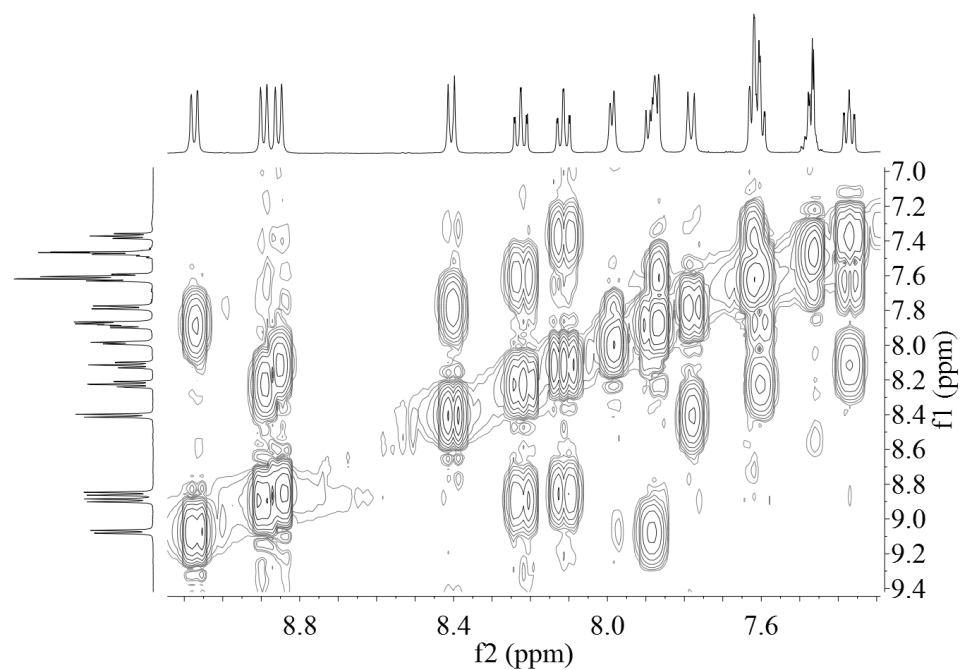


C


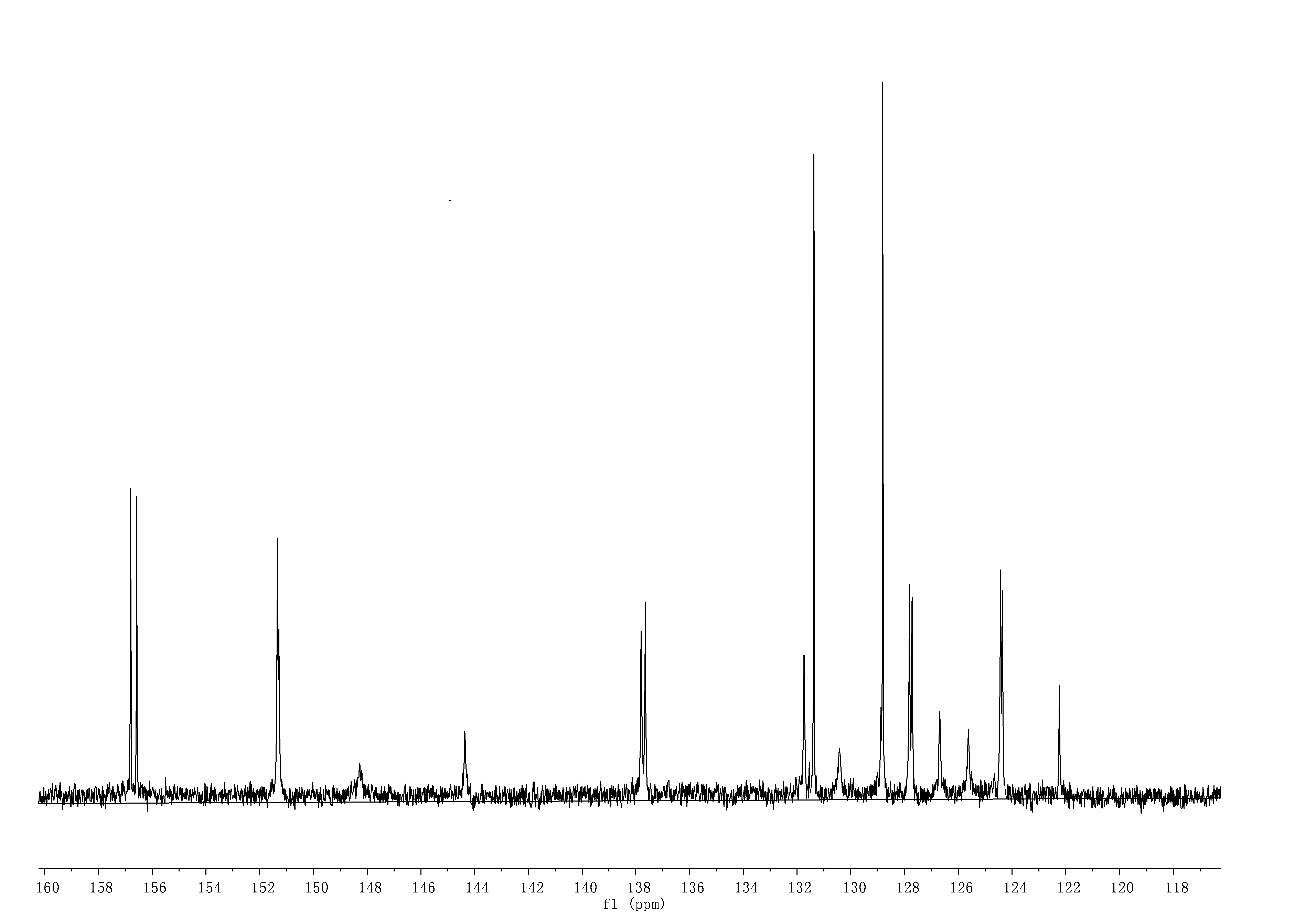


D

E

F

**Figure S1**. The characterization data of chiral ruthenium complex ***Λ*-RM0627**. (A) The ESI-MS spectra of ***Λ*-RM0627**; (B) The ^1^H NMR spectra of ***Λ*-RM0627**; (C) The ^1^H ^1^H COSY spectra of ***Λ*-RM0627**; (D) The ^13^C NMR spectra of ***Λ*-RM0627**; (E) The elctronic absorption spectra of ***Λ*-RM0627**; (F) The CD spectra of ***Λ*-RM0627**.

A B

**Figure S2**. (A) The fluorescence spectra of *Λ-*RM0627 with the addition of the distilled water. ([Ru] = 20 μ M, V_(H2O)_ = 2n μL; n =0, 1, 2, etc.); (B) The fluorescence spectra of FITC*-c-myc* DNA with the increasing of the DNA concentration. ([DNA] = n μ M, μL; n =0, 1, 2,...10).


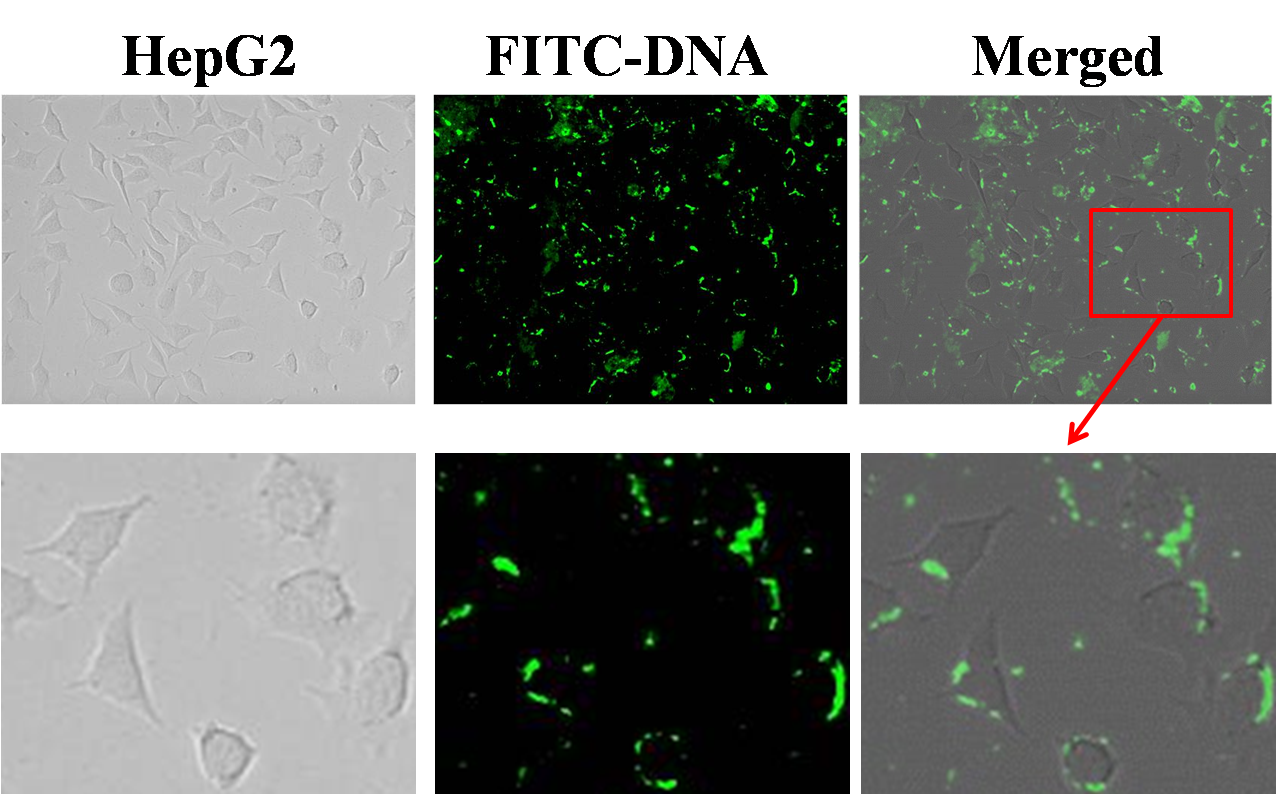


**Figure S3**. The cellular uptake of FITC-DNA in HepG2 cells. The cells were incubated by FITC-DNA (5 μM) for 6 h**,** then cells were washed 3 times by PBS and fixed with 70% ethanol. The pictures were captured by fluorescence microscope.

**Figure S4**. The cellular locasition of ***Λ-*RM0627** in HepG2 cells. The cells were incubated by***Λ-*RM0627** (5 μM) for 6 h**,** then cells were washed 3 times by PBS and stained with DAPI .


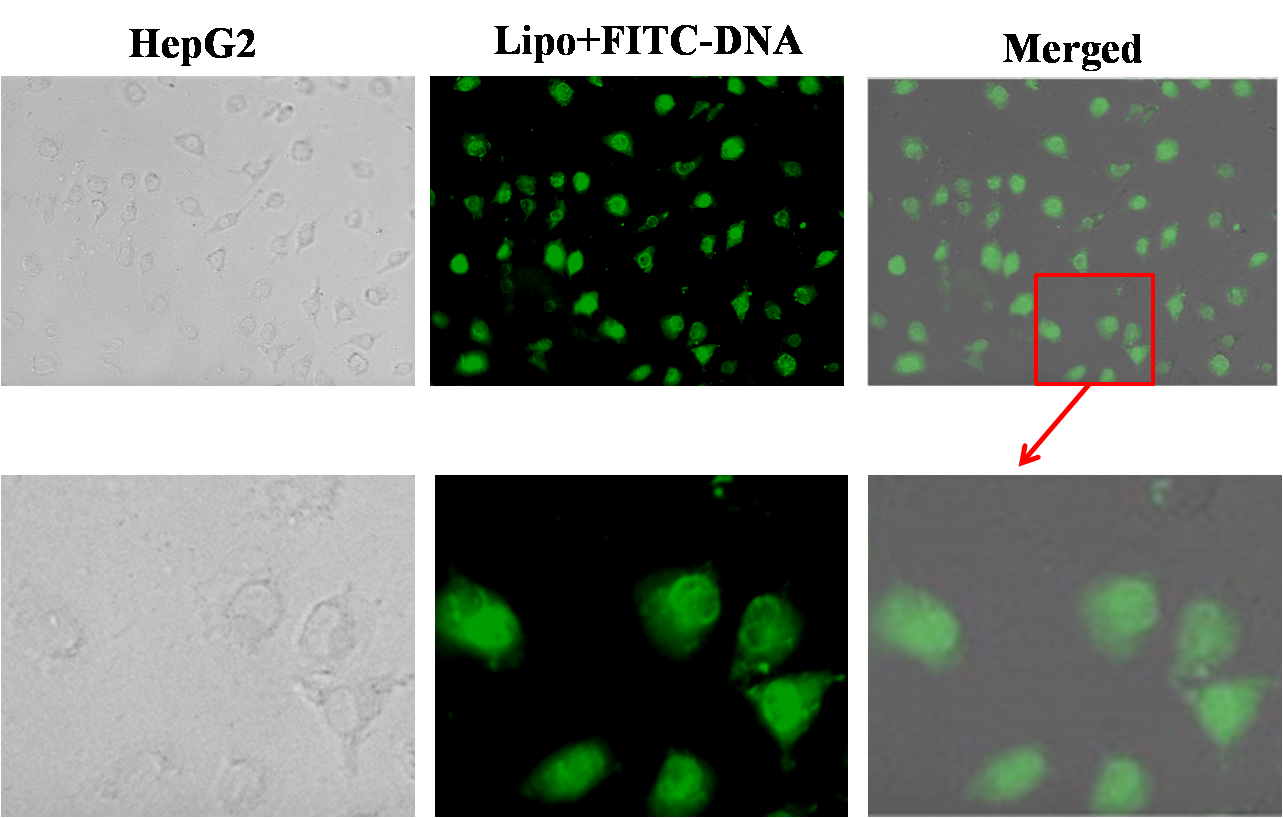


**Figure S5**. The transportation of FITC-DNA delivered by lipo 2000 in HepG2 cells. The cells were incubated by FITC-DNA (5 μM) and lipo 2000 (2 μL) for 24 h**,** then cells were washed 3 times by PBS and fixed with 70% ethanol. The pictures were captured by fluorescence microscope.


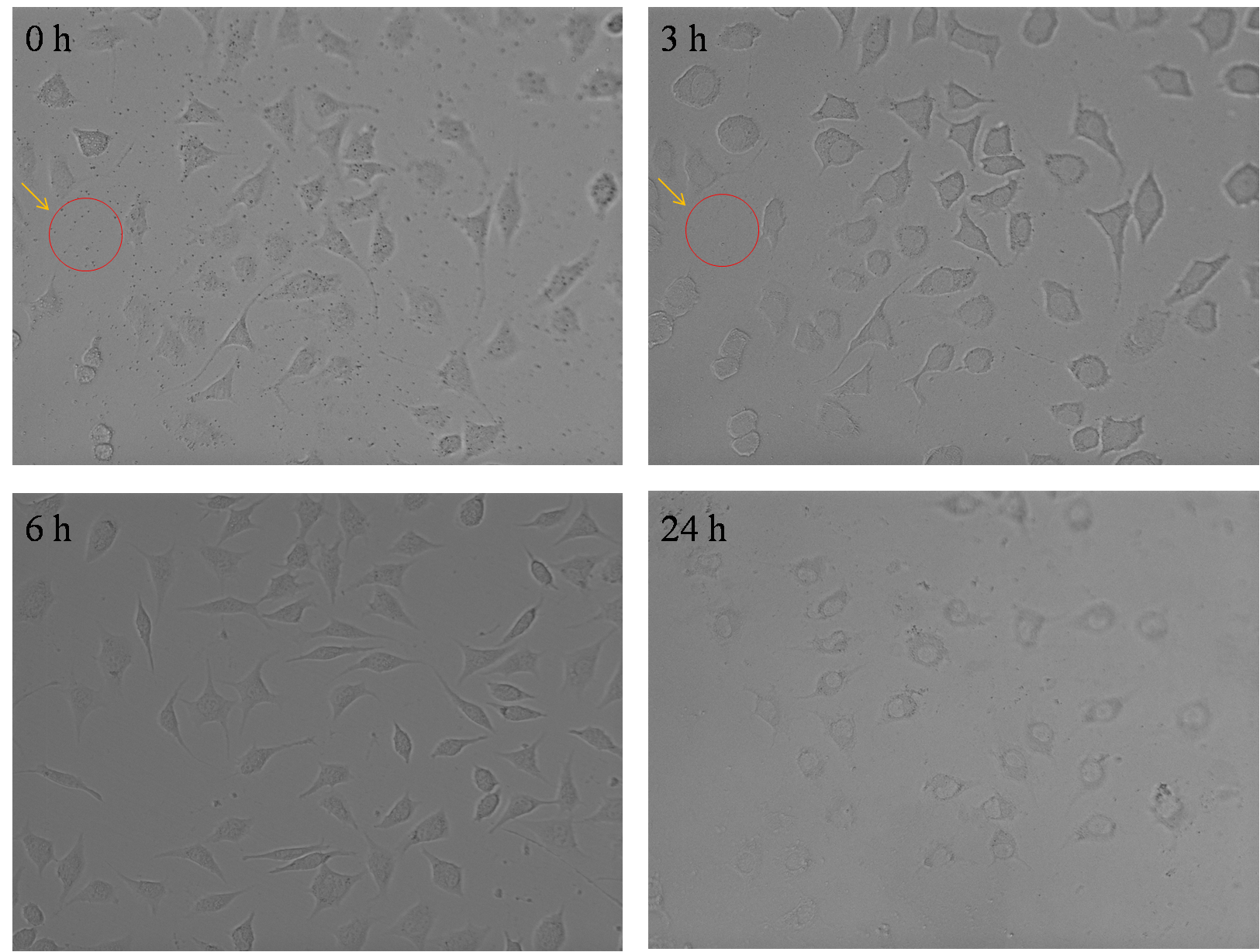


**Figure S6**. The morphologic observation of HepG2 cells treatment with lipo 2000 **(**50 μL**)** in 2 mL DMEM for 24 h. In the three previous hours, the cells are incubated in live cell imaging with 5% CO_2_ for 37 ^o^C, and the pictures were captured at the same view by fluorescence microscope. After three hours the pictures were captured at different view by fluorescence microscope.

# 3. REFERENCES

1 Ou, T. M. *et al.* Stabilization of G-quadruplex DNA and down-regulation of oncogene c-myc by quindoline derivatives. *J. Med. Chem.* **50**, 1465-1474 (2007).

2 Zhong, H., Wang, J., Li, L. & Wang, R. The copper-free Sonogashira cross-coupling reaction promoted by palladium complexes of nitrogen-containing chelating ligands in neat water at room temperature. *Dalton Trans.* **43**, 2098-2103 (2014).

3 Baggaley, E. *et al.* Dinuclear ruthenium(II) complexes as two-photon, time-resolved emission microscopy probes for cellular DNA. *Angew. Chem. Int. Ed.* **53**, 3367-3371 (2014).

4 Gill, M. R. *et al.* A ruthenium(II) polypyridyl complex for direct imaging of DNA structure in living cells. *Nat. Chem.* **1**, 662-667 (2009).

5 Catherall, T., Huskisson, D., McAdams, S. & Vijayaraghavan, A. Self-assembly of one dimensional DNA-templated structures. *J. Mater. Chem. C* **2**, 6895-6920 (2014).

6 Yatsunyk, L. A. *et al.* Guided assembly of tetramolecular G-quadruplexes. *ACS Nano* **7**, 5701-5710 (2013).

7 Hessari, N. M. *et al.* Programmed self-assembly of a quadruplex DNA nanowire. *Chemistry* **20**, 3626-3630 (2014).

8 Rudiuk, S., Venancio-Marques, A., Hallais, G. & Baigl, D. Preparation of one- to four-branch silver nanostructures of various sizes by metallization of hybrid DNA-protein assemblies. *Soft Matter* **9**, 9146-9152 (2013).

9 Liu, L. *et al.* Dinuclear metal(II) complexes of polybenzimidazole ligands as carriers for DNA delivery. *Biomaterials* **31**, 1380-1391 (2010).
